# Dienogest versus norethisterone acetate in management of endometrial hyperplasia without atypia

**DOI:** 10.1007/s00404-023-07015-7

**Published:** 2023-04-03

**Authors:** Ehab F. Girbash, Hala E. Sherif, Ahmed M. Radwan, Hussein M. Abdeldayem

**Affiliations:** Obstetrics and Gynecology Department, Faculty of Medicine, Zagazig University, Zagazig, Egypt

**Keywords:** Endometrial hyperplasia without atypia, Dienogest, Norethisterone acetate

## Abstract

**Objectives:**

To compare the effectiveness of dienogest (DIE) and norethisterone acetate (NETA) regimens in the treatment of endometrial hyperplasia (EH) without atypia.

**Methods:**

Participants were premenopausal women with irregular uterine bleeding, and endometrial hyperplasia without atypia on endometrial biopsy. Enrolled patients were randomly allocated into two groups: group I got DIE 2 mg/day (orally Visanne) for 14 days (10th to the 25th day of cycle) while group II received between the 16th and 25th day of the cycle, norethisterone acetate (NETA) 15 mg/d (orally Primolut Nor) was administered for 10 days. Both groups continued the therapy for six months.

**Results:**

The DIE group showed a higher resolution (32.7%) and regression (57.7%) than NETA group (31% & 37.9%, respectively) with significant regression (*p* = 0.039). No progression in DIE group while four (6.9%) women in NETA group were recorded a progression to complex type without a significance. Also, NETA group showed a significant persistence rate (22.5%) than DIE group (3.8%) (*p* = 0.005). Also number in NETA group managed by hysterectomy with significant difference (*p* = 0.042).

**Conclusion:**

If used as first-line treatment, Dienogest produces a better rate of regression and a lower incidence of hysterectomy than Norethisterone Acetate does when used in EH without atypia.

## What does this study add to the clinical work

**Table:** 

Norethisterone Acetate can be used as first-line management in endometrial hyperplasia without atypia. A lower incidence of hysterectomy found.	
Dienogest produces a better rate of regression and safety and a lower incidence of hysterectomy than Norethisterone Acetate does when used in endometrial hyperplasia without atypia. So can be used as first-line management.	

## Introduction

Endometrial hyperplasia, which may lead to endometrial cancer or coexist with it, is characterized by an excessive growth of the endometrial glands [1]. The primary factor causing endometrial hyperplasia is an elevated estrogen to progesterone ratio [2].

Although this ailment is often detected during the postmenopausal stage, it may occur at any age as long as the person is exposed to estrogen. The most significant risk factor for the development of endometrial cancer from hyperplasia is hyperplasia with cellular atypia. The most prevalent indication of endometrial hyperplasia is irregular uterine hemorrhage [3].

In 80% of instances, simple hyperplasia that is left untreated heals on its own; in 19% of cases, it persists; and in 1% of cases, it turns into cancer. However, the chance of developing cancer rises when cytologic atypia is present [4].

The progestin's mode of action for treating hyperplasia involves pseudo-decidualization, a decrease in the number of endometrial glands, and their anti-estrogenic activities [5]. The majority of individuals with hyperplasia react to the progestin medication and do not develop cancer; however, those who do not react to progestins have cytologic atypia and a high risk of developing cancer [6]. Unusual hyperplasia and well-differentiated endometrioid cancer may both be successfully treated with progestin medication in women under the age of 40 [7].

Progestin is proven to be useful in treating endometrial hyperplasia, however the side effects of progestin medications, particularly older second-generation medications, include weight gain, mood changes, acne, amenorrhea, and negative vascular consequences. [8, 9].

Dienogest (DIE), a fourth-class oral progestin from a new generation, combines the pharmacologic characteristics of progestin derivatives with those of 19-norprogestins. It has potent progestogenic impacts without androgenic, mineralocorticoid, or glucocorticoid activities, making it particularly useful for the cure of endometriosis [10]. Shimizu et al. [11] revealed that for gynecological conditions linked to aberrant endometrial proinflammatory and proliferative activity, DIE may be regarded as a helpful therapy [12]. In another research, Kodama et al. [13] revealed that the effectiveness of DIE for endometrial thinning prior to hysteroscopic surgery. They demonstrated that the endometrium shrunk after receiving DIE (2 mg orally daily) for two weeks. Consequently, we proposed the possibility that DIE may be utilized to treat EH.

Our research compares the efficacy of DIE and norethisterone acetate (NETA) regimens for treating EH without atypia.

## Patients and methods

### Study design and inclusion criteria

**Fig. 1:**
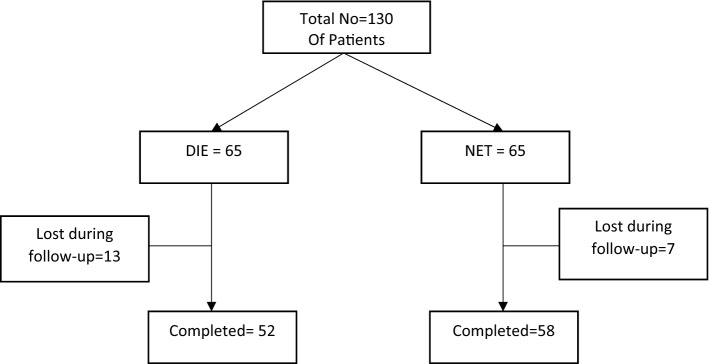
Flow chart of studied patients

A prospective controlled study of premenopausal women between the ages of 41 and 53 who were hospitalized to the Obstetrics and Gynecology Department at Zagazig University between January 2020 and October 2021 with abnormal uterine hemorrhage and persistent pelvic discomfort participated in an interventional prospective study (Fig. 1). According to the Institutional Review Board of Zagazig University Faculty of Medicine, all subjects gave written, informed permission to take part in the research.

Participants in the research had abnormal endometrial thickness (more than 12 mm) on transvaginal ultrasonography and endometrial hyperplasia without atypia on endometrial biopsy (according to the WHO endometrial hyperplasia criteria) [14].

### Exclusion criteria

However, those with localized endometrial lesions like leiomyoma or atypical or complicated hyperplasia who have had hormone treatment within the last six months are at higher risk and other causes of abnormal uterine bleeding (AUB) like adnexal swellings and genital and breast cancers were excluded. Also, patients with liver, kidney, heart, diabetes, thromboembolic diseases or steroid therapy were ineligible.

**Fig. 2:**
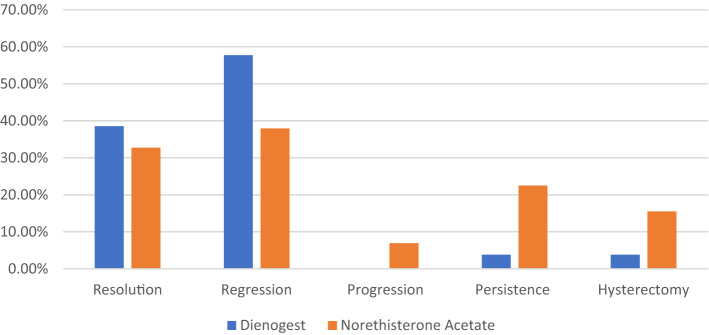
Outcomes of therapeutic treatment of EH for six months

### Interventions

Enrolled patients were randomly allocated into two groups; group I got DIE 2 mg/day (orally Visanne; bayer pharma, Cairo, Egypt) for 14 days (10th to the 25th day of cycle) while Between the 16th and 25th day of the cycle, group II got norethisterone acetate (NETA) 15 mg/d (orally Primolut Nor; Hi Pharma, Cairo, Egypt) for 10 days. Both groups continued the therapy for six months.

A list of random numbers created by a computer was used for randomization. The QuickCalcs program was used to create the random number list (GraphPad Software Inc, La Jolla, California). The research supervisor stored the group assigning numbers in an envelope that was sealed.

Clinical evaluation and history-taking are performed on all women. In addition, a transvaginal ultrasound was used, and women with abnormal endometrial thickness underwent biopsy using the Pipelle Endometrial Suction Curette (Cooper Surgical Inc., Trumbull, CT 06,611 USA). The Pipelle is a single-use, sterile, plastic, disposable, suction curette consisting of an outer graded sheath and an inner piston for obtaining a histologic biopsy of the uterine mucosa lining or sample extraction of uterine menstrual content for microscopic examination or culturing. The Pipelle had outer diameter (OD) of 3.1 mm and no cervical dilatation needed. The biopsy sample was then examined by the unit of pathology. All women followed-up for six months then the second biopsy and histopathology were done.

The pathologic outcomes of endometrial curettages were compared (both during and after therapy, respectively). The outcomes were divided into four groups: advancement (simple EH without atypia), persistence (atrophic, inactive, secretory endometrium), regression (proliferative endometrial) (simple EH atypia, complicated EH atypia, or neither).

### Statistical analysis

On an IBM compatible computer, the acquired data were tabulated and analyzed utilizing SPSS (statistical software for social research), version 25 (IBM, Armonk, NY, USA). Quantitative continues group represents by mean ± SD and compared between them by independent t test and paired t test while qualitative represents as number and percentage and is compared between them utilizing chi square test. *P* value < 0.05 was considered substantial.

## Results

A total of 110 women with a typical EH divided into two therapeutic group (DIE & NETA), their mean of age was 43.21 ± 8.15 and 45.83 ± 6.44, respectively. There was no significance between groups considering age, BMI, comorbidity and parity. The most frequent pattern of AUB in DIE & NETA groups was menometrorrhagia and 21 (40.4%) and 18 (31%) of the patients had complained from it. There was no significance between groups considering types of AUB (Table 1).

**Table 1:** Demographic data of studied women

	Dienogest (*n* = 52)	Norethisterone acetate (*n* = 58)	*t*/*x*^2^ test	*P* value
Age (year)	43.21 ± 8.15	45.83 ± 6.44	1.880	0.063
BMI kg/m^2^	28.37 ± 2.17	27.52 ± 3.52	−1.504	0.136
Comorbidity				
HTN	7 (13.5%)	5 (8.6%)	0.671	0.413
D.M	4 (7.7%)	6 (10.3%)		
Cardiac diseases	1 (1.9%)	2 (3.4%)		
Parity				
Nulli	16 (30.8%)	21 (36.2%)	0.355	0.551
1	29 (55.8%)	27 (46.5)		
> 2	7 (13.4%)	10 (17.3%)		

*BMI* body mass index, *HTN* hypertension, *D.M* diabetes meletus

Regarding histological type of EH in DIE & NETA groups, simple type present in 19 (36.5%) and 22 (37.9%) of women while complex type present in 33 (63.5%) and 36 (62.1%) of women without significance. Also, endometrial thickness by vaginal ultrasound was comparable between both groups (Table 2).

**Table 2:** Comparison between groups regarding histological type of EH and endometrial thickness

	Dienogest (*n* = 52)	Norethisterone acetate (*n* = 58)	*t*/*x*^2^ test	*P* value
Histological Types				
Simple	19 (36.5%)	22 (37.9%)	*x*^2^ = 0.023	0.88
Complex	33 (63.5%)	36 (62.1%)		
Endometrial thickness (mm)	16.51 ± 3.49	17.27 ± 4.11	*t* = 1.039	0.301

Considering outcome, DIE group showed a higher rate of resolution (32.7%) and regression (57.7%) than NETA group (31% & 37.9%, respectively) with significance regarding regression only (*p* = 0.039). There was no patient showed progression in DIE group while 4 (6.9%) women in NETA group were recorded a progression to complex type without a significance. Also, NETA group showed a higher significant persistence rate (22.5%) than DIE group (3.8%) (*p* = 0.005). Two women in DIE group and nine women in NETA group who showed persistence of complex EH and complete their family managed by hysterectomy with significant difference (*p* = 0.042). Statistically significant result was at a P-value of ≤ 0.05 (Table 3) (Fig. 2).

**Table 3:** Outcomes of therapeutic treatment of EH for six months

	Dienogest (*n* = 52)	Norethisterone acetate (*n* = 58)	*x*^2^ test	*P* value
Resolution	20 (38.5%)	19 (32.7%)	0.399	0.527
Regression	30 (57.7%)	22 (37.9%)	4.273	0.039*
Progression	0 (0%)	4 (6.9%)	3.690	0.055
Persistence	2 (3.8%)	13 (22.5%)	8.056	0.005*
Hysterectomy	2 (3.8%)	9 (15.5%)	4.144	0.042*

There was a significance between both groups considering endometrial thickness after intervention (*p* = 0.047). Also, there was a significance between endometrial thickness before and after intervention in both groups (*p* < 0.0001). Statistically significant result was at a P-value of  ≤ 0.05 (Table 4).

**Table 4:** Comparison between endometrial thickness before and after intervention in both groups

	Dienogest (*n* = 52)	Norethisterone acetate (*n* = 58)	*t* test	*P* value
Endometrial thickness before intervention	16.51 ± 3.49	17.27 ± 4.11	1.039	0.301
Endometrial thickness after intervention	7.93 ± 3.16	9.44 ± 4.53	2.005	0.047*
*P* value (paired *t* test)	< 0.0001**	< 0.0001**		

Side effects of DIE & NETA were comparable between groups with slightly higher rate in NETA group for irregular bleeding, nausea &vomiting and mastalgia (Table 5).

**Table 5:** Distribution of side effect of DIE and NETA in studied women

	Dienogest (*n* = 52)	Norethisterone acetate (*n* = 58)	*x*^2^ test	*P* value
Irregular bleeding	1 (1.9%)	3 (5.2%)	0.844	0.358
Hot flushes	1 (1.9%)	1 (1.7%)	0.006	0.937
Nausea and vomiting	1 (1.9%)	2 (3.4%)	0.233	0.629
Dizziness	0 (0%)	1 (1.7%)	0.884	0.347
Mastalgia	1 (1.9%)	2 (3.4%)	0.233	0.629

## Discussion

Endometrial hyperplasia is a widespread condition. Particularly in Arab nations, the uterus is seen as a sign of women and femininity. Her psychological state will be greatly impacted by having her uterus removed. Therefore, every effort must be made to maintain the uterus as much as possible.

### Main findings

To the extent that we are aware, there is insufficient data regarding effect of DIE in EH without atypia and our study is a unique one to compare its effect versus NETA as a conventional treatment of EH.

### Strengths and limitations

The main limitation of research was the small size of study groups, and the factors strengthen the study, the prospective nature of study and use of inclusion and exclusion criteria with coding of the data collected for proper interpretation.

### Interpretation

A fourth-generation progestin called DIE was employed in hormone replacement treatment and contraceptive pills [15]. Sasagawa et al. [12] showed that compared to other progestins, DIE showed a strong selectivity to the progesterone receptor. In addition, compared to other progestins at greater dosages, DIE exhibited the highest endometrial action and higher plasma concentrations. DIE is also advised as a first-line therapy for endometriosis that causes pelvic pain because it has been shown to be highly efficient in the treatment of endometriosis [16].

It is well known that progestins speed up the signaling pathways that prevent endometrial cells from undergoing cancer development [17]. H However, there is disagreement on the best form of progestin. In addition, the mechanism of progestin’s anticancer effect is attracting more and more attention in research.

A research by Kodama et al. [13] revealed that the effectiveness of using DIE to thin the endometrium prior to hysteroscopic surgery, and they also assessed the results of the procedure. 26 individuals had endometrial polyps or submucous myomas that were identified by ultrasonography as having a thick endometrium (The largest endometrial diameter was under 25 mm.). Before hysteroscopic surgery, 13 patients got a gonadotropin-releasing hormone agonist subcutaneously 1–3 times every 4 weeks and 13 individuals took 2 mg DIE orally twice a day for a mean of 14.0 days. Twelve patients from each group had surgery with endometrium that was both thinned and easily visible. Identical to this research, Cicinelli et al. [17]. In patients treated with estradiol with DIE, hysteroscopic polypectomy was conducted on an endometrium that was thinner and well-prepared. Both investigations highlighted the fact that DIE is a particular progesterone receptor agonist with a potent inhibitor of endometrial proliferation, improved patient compliance, and less hazards.

Ozdegirmenci et al. [18] examined the use of several progestins, such as MPA (10 mg/day), lynestrenol (15 mg/day), and norethisterone (15 mg/day) for 10 days each cycle, in the treatment of uncomplicated EH without atypia. They asserted that at such doses, the efficacies of the three progestins previously stated were comparable in uncomplicated EH without atypia. However, their findings did not establish whether one progestin was better than another or vice versa.

In the current study, we used DIE 2 mg/day for 14 days/cycle orally versus NETA 15 mg/day for 10 days/cycle orally in patients with EH without atypia for six months. We found that DIE group showed a higher rate of resolution (38.5%) and regression (57.7%) than NETA group (31% & 37.9%, respectively) with significance regarding regression only (*p* = 0.039). There was no patient showed progression in DIE group while 4 (6.9%) women in NETA group were recorded without significance. Also, NETA group showed a higher significant persistence rate (22.5%) than DIE group (3.8%) (*p* = 0.005). In addition to acting like progestin, DIE also suppresses the expression of aromatase and PGE2 synthesis in human endometrial epithelial cells [11]. Additionally, DIE's suppressive impact on cyclin D1 levels helped to limit the growth of human immortalized endometrial epithelial cells [18]. Therefore, we propose that the outcome of our research may be explained by the direct inhibitory and antiproliferative actions.

The same results reported by Uysal et al. [19]. The greatest rates of resolution and regression were seen in the DIE group, despite the fact that there was no statistically significant difference between the groups (*p* = 0.39). This study compared the effects of DIE, MPA, and MP in EH without atypia. Ismail et al. [20] reported that a resolution rate of EH without atypia was 40% in patients treated by NETA, regression rate of 56.67% and persistence occurred in one patient.

Also, our results revealed that side effects of DIE & NETA were comparable between groups with slightly higher rate in NETA group for irregular bleeding, nausea &vomiting and mastalgia. The same finding recorded in Uysal et al. [19] study who showed that side effects were similar between DIE, MPA and MP groups. However, other research has shown that DIE has a decreased propensity for venous thrombosis risk [21, 22]. This may be due to the short follow-up period of our study which we considered it as a limitation of our work.

## Conclusion

If used as first-line treatment, Dienogest produces a better rate of regression and a lower incidence of hysterectomy than Norethisterone Acetate does when it comes to thinning the endometrium in EH without atypia.

## Data Availability

Data available on reasonable request.
